# Granulomatous Invasive *Aspergillus flavus* Infection Involving the Nasal Sinuses and Brain

**DOI:** 10.1177/2324709618770473

**Published:** 2018-05-02

**Authors:** Roopam Jariwal, Arash Heidari, Ahana Sandhu, Janushe Patel, Kamelia Shoaepour, Piruthiviraj Natarajan, Everardo Cobos

**Affiliations:** 1 Kern Medical Center, Bakersfield, CA, USA

**Keywords:** granulomatous invasive *Aspergillus flavus*, immunocompetent host, Indian origin, rhinosinusitis, voriconazole, skull and retro-orbital structures, maxillary sinus

## Abstract

Invasive fungal infections are commonly associated with some form of immunosuppression. On the nasal epithelial surface, *Aspergillus flavus*, under favorable conditions, can aggressively breach multiple cell lines invading the local tissues. We present the case of a 35-year-old woman with granulomatous invasive *Aspergillus flavus* infection involving the nasal sinuses and the brain. Antifungal agents administered in the previous episodes contained the infection; however, the infected site evolved over time surrounded with calcified tissues in the left maxillary sinus. The current infection involved the other side of the maxillary sinus and extended to the orbital cavity eroding the parts of the skull and retro-orbital structures and was treated with a long course of isavuconazole therapy.

## Introduction

Fungal colonization of nasal air passages is common among humans with frequent exposure to the *Aspergillus flavus* fungal conidia from the environment. Commonly, *Aspergillus* fungi are found to coexist with other microorganisms in the skin, vagina, lungs, intestines, and oral cavity.^1^ An extreme high exposure to *Aspergillus* fungal conidia can promote the fungal infection in an immunocompetent host.^2^ The incidence of *Aspergillus* infection and its associated mortality has increased on administration of recent immunosuppression therapies.^3,4^ The transition to infect the host depends on the host immune status and nasal tight junction breach, which enables the hematogenous access to the fungi. Invasive *Aspergillus flavus* rhinosinusitis is uniquely reported more in northern part of India.^5,6^

## Case Presentation

**Figure 1.**
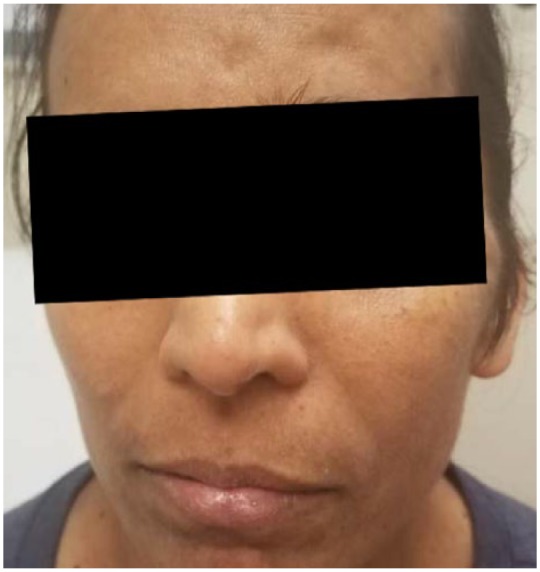
Left facial swelling on presentation.

A 35-year-old Indian woman presented at our emergency department with left facial pain and swelling (Figure 1) for 2 months associated with intermittent frontal headache for the past 4 months. Previously, she had left nasal polyps that were removed twice in the years 2002 and 2011. She had an episode of aspergillosis of the left maxillary sinus invading the left frontal lobe, which was removed surgically in the year 2005. After receiving voriconazole therapy for the next 25 months she recovered from her symptoms.

Physical examination showed an incompressible firm round mass behind the left temple region. An irregular indentation over the left forehead area of 3 × 4 cm size and scars along the temporal region were noticed.

**Figure 2.**
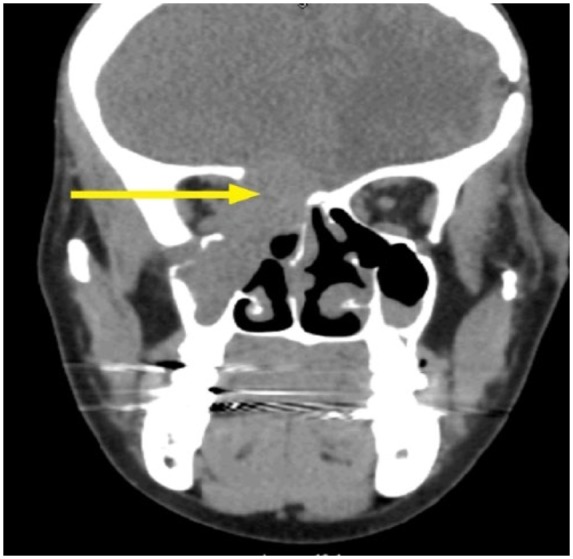
Computed tomography scan of the brain and maxillary sinus mass with bony erosion of the cribriform plate.

**Figure 3.**
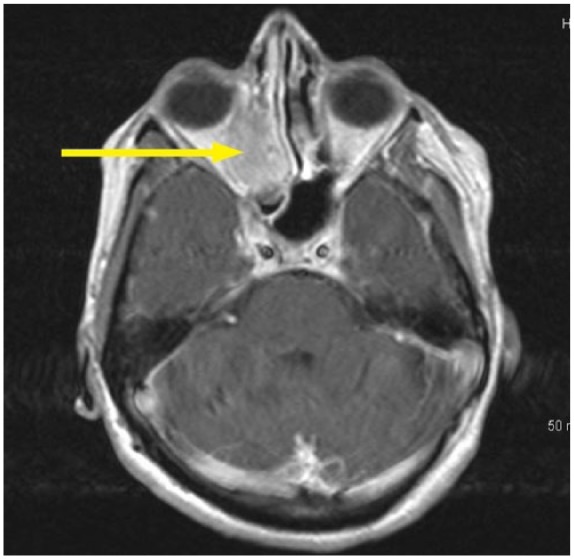
Magnetic resonance imaging right retro-orbital area with the suspected fungal mass.

**Figure 4.**
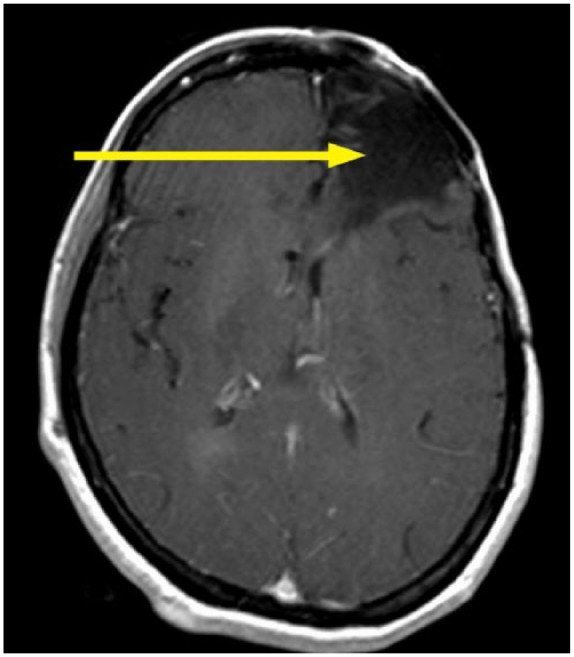
Left frontal encephalomalacia due to surgical removal of the frontal lobe with prior aspergillosis.

Computed tomography (Figure 2) and magnetic resonance imaging scan of the head revealed a soft tissue mass measuring 3.8 × 2.8 cm in the right maxillary sinus with bone destruction and displacement of the medial rectus muscle (Figure 3). The mass eroded through the cribriform plate and involved the pachymeningeal layers. There was an additional left maxillary sinus mass measuring 4 cm with bone destruction. Left frontal lobe encephalomalacia (Figure 4) was attributed to the frontal lobe resection during the previous episode.

**Figure 5.**
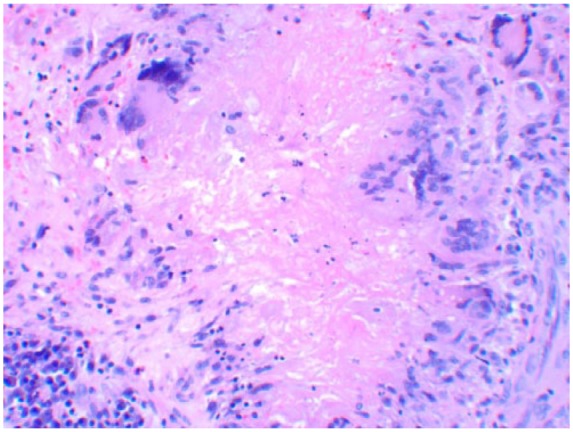
Irregular borders with central necrosis.

**Figure 6.**
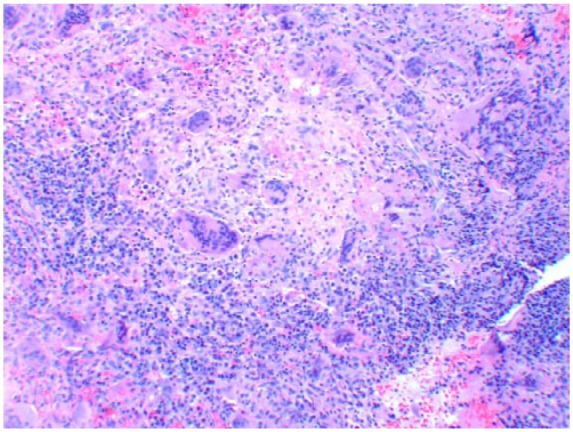
Necrotizing granulomatous inflammation surrounded by lymphocytes.

**Figure 7.**
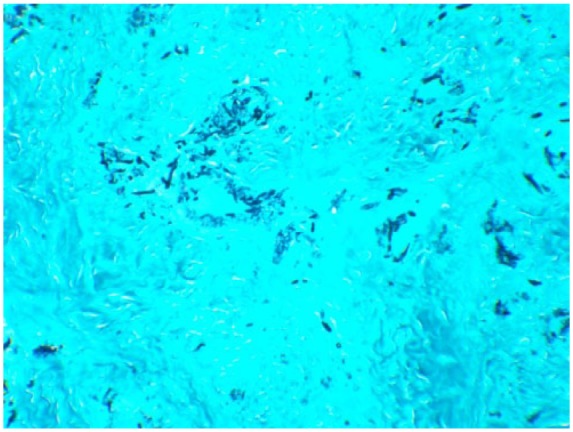
Grocott’s methenamine silver stain showed septate hyphae with dichotomous branching.

**Figure 8.**
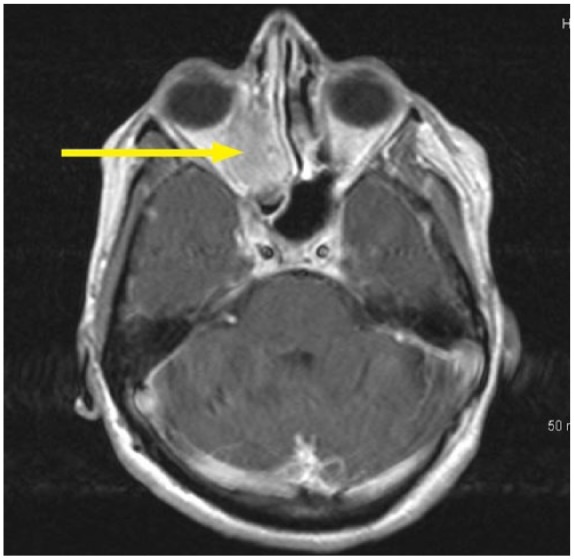
Magnetic resonance imaging right retro-orbital area after 6 months of isavuconazole treatment.

Nasal endoscopy revealed a missing middle turbinate on the left side. The left maxillary sinus contained a dark gray-colored fluid with calcified fungal debris. Biopsy of the tissues showed necrotizing granulomatous inflammation (Figure 5) with multinucleated giant cells and eosinophils surrounding the lesions (Figure 6). Nasal sinus fluid cultures were positive for *A flavus* with minimum inhibitory concentration of 0.25 µg/mL for isavuconazole and voriconazole with 0.125 µg/mL for posaconazole. The decision to choose isavuconazole was made since the patient had no insurance and was enrolled in a patient-assisted pay program that provided funding for isavuconazole. Grocott’s methenamine silver stain of the maxillary sinus mass revealed septate hyphae with dichotomous branching *Aspergillus* (Figure 7). Serum total immunoglobulin E level was 1521 kU/L (normal ≤127 kU/L). She did not have measurable immune deficiency. A repeat magnetic resonance imaging performed 6 months later showed reduction in the size of the mass (Figure 8). Within 2 months of isavuconazole treatment, she is able to appreciate the sense of smell, which was not possible earlier. She is planned to receive a lifelong course of isavuconazole to avoid recurrence of the disease.

## Discussion

Fungal organisms constitute 0.1% of the entire nasal microbiota spectrum.^7^ Following the exposure to airborne *A flavus* conidia, the fungal particles settle on an intact nasal mucosal surface and maintains a symbiotic coexistence. The larger *A flavus* (3-5 µm) are mostly filtered at the upper respiratory structures, whereas the much smaller *A fumigatus* (2.5-3.5 µm) reach the alveoli.^8^ Most of the microorganisms are brushed off by the upper respiratory tract by an intact ciliated columnar epithelium, whereas a few could still endure on the mucosal surface. Only a breach at the tight junctions of the nasal mucosal layers can enable a fungal invasion. In a hypersensitive host, an associated frequent fungal particle exposure results in an episodic inflammatory battleground scenario at the nasal mucosal layers. The raised interleukin-5 levels and activated eosinophils in the mucin directly damage the epithelial barrier enabling access to the fungi and bacterial organisms.^9^ The conidial exposure, associated hypersensitivity, secondary upper respiratory infection, and the resulting altered microbiota becomes a vicious cycle. Aflatoxins secreted by *A flavus* reduce mucociliary action at the nasal mucosal surface.^10^ Following the entry, the fungal particles express their elastase^11^ and hydrolase activity on the mucosal layers aiding their survival.^12^ Additionally, complement system inactivation by binding to the complement regulating factors to evade the immune mechanism has been noticed in *A fumigatus*.^13^ In an immunocompetent host the immune system responds to the β-D-glucan component of the fungal cell wall to recruit acute inflammatory cells. The local dendritic cells and macrophages can ingest fungal particles and present it to T-helper cells at the lymph nodes for destruction of the fungi. The deficiency in certain toll-like receptors of the host immune system that recognize β-D-glucan and other components of the fungi can prevent immune recognition of the fungi. A mutation in one of the genes (S100, SPINK-5) coding for the membranes involved in barrier function of the nose makes the nasal mucosal surface more vulnerable.^14^ Assessing for common immune deficiency diseases when diagnosed with invasive fungal infections is not currently a standard of care but it could be useful in recurrent invasive infections.

The histological examination of the calcified tissues from the maxillary sinus suggest a chronic infection core surrounded by calcified inflammatory cells. A superimposed infection in the adjacent nasal mucosa recruited the inflammatory mediators to the site created a necrotic sludge of dead tissues. The development of frequent nasal polyps is associated with *A flavus* rhinosinusitis.^15^ High-efficiency particulate air filtration could reduce the fungal exposure load when exposed to dust or dust-generating activities. It is imperative to review the patient for the side effects of long course antifungal agents and be watchful for a suspected aspergillosis reinfection.

## Conclusion

The incidence of aspergillosis has increased multiple folds in immunocompromised hosts. Even in an immunocompetent host, granulomatous invasive *A flavus* rhinosinusitis is a life-threatening condition, and clinical suspicion should be high in patients with recurrent fungal rhinosinusitis and/or nasal polyps especially with those of Indian origin. Clinical aggressive measures to remove the fungal mass and the specific antifungal agents could prevent the local invasion to the brain and the subsequent associated mortality.
